# Use of Helical TomoTherapy for the Focal Hypofractionated Treatment of Limited Brain Metastases in the Initial and Recurrent Setting

**DOI:** 10.3389/fonc.2015.00027

**Published:** 2015-02-09

**Authors:** Andrew Elson, Ashley Walker, Joseph A. Bovi, Christopher Schultz

**Affiliations:** ^1^Department of Radiation Oncology, Medical College of Wisconsin, Milwaukee, WI, USA

**Keywords:** brain metastases, radiotherapy, fractionated SRS, TomoTherapy, hypofractionated radiotherapy, oligometastases

## Abstract

**Background**: Whole-brain radiation therapy (WBRT), stereotactic radiosurgery (SRS), or both are commonly employed in the treatment of limited brain metastases in the initial or recurrent setting. Hypofractionated partial volume irradiation is also employed, however, published experience using helical TomoTherapy (HT) for this purposes is limited. We reviewed our institutional experience to assess patient selection factors, fractionation scheme, and outcomes associated with this technique.

**Methods:** A retrospective chart review was performed to evaluate patients treated with partial volume hypofractionated HT-based IMRT for brain metastases at our institution.

**Results:** Thirteen patients (7M/6F, median age 62, median KPS 90) with a limited (1–9) number of brain metastases in the primary or recurrent setting were identified. Primary malignancies included colorectal (3), NSCLC (5), RCC (1), breast (1), melanoma (1), uterine (1), and ovarian (1). The median time from initial diagnosis to brain metastases was 20.7 months (range 0–61.3). Treatment was delivered to intact metastases in six patients, to a single resection cavity in six patients, and to both in one patient. A total of 27 lesions were treated. The median number of intact metastases treated was two (range 1–9). Previous treatments included WBRT (5), WBRT + SRS (3), SRS alone (1), and none (4). The most common fractionation schemes were 25 Gy in five fractions and 27.5 Gy in five fractions to each lesion. At a median of 6 months follow up (range 1.26–20.13) after TomoTherapy, 10 patients were deceased, 2 were alive, and 1 was lost to follow up. Systemic progression occurred in seven patients and intracranial progression occurred in five. The median intracranial progression free survival and overall survival after TomoTherapy was 6.3 months. Freedom from local failure for treated lesions was 71% and 59% at 6 and 12 months.

**Conclusion:** TomoTherapy-based hypofractionated radiotherapy to a limited number of metastatic lesions is associated with acceptable intracranial disease control and survival outcomes and represents a viable treatment option in the primary and recurrent setting for select patients.

## Introduction

Brain metastases as a systemic manifestation of cancers from various primary sites and histologies has historically implied an extremely poor prognosis with survival measured in weeks in untreated cases (1). Traditionally, the use of whole-brain radiation therapy (WBRT) has been the mainstay of treatment for brain metastases with an improvement in survival estimated on the order of several months (2). Improvements in the care of patients with metastatic disease in several domains including advances in systemic therapies such as combination chemotherapy and targeted agents, improvements in control of the primary lesion, and advances in radiotherapeutic modalities has resulted in a proportion of patients surviving with brain metastases for a longer duration (3). In addition, the stratification of patients according to prognostic factors including age, performance status, and control of primary disease has attempted to identify patients with potentially durable survival (4). The concept of “oligometastatic” brain disease, which is variably defined, but often on the order of 1–5 brain metastases, has led to alternative management considerations other than the routine application of WBRT as a sole modality (5, 6). Given that reports have described select patients with brain metastases with survival on the order of several years, the management of oligometastatic brain disease reflects a balance between optimal intracranial disease control and potential toxicities or adverse long term sequelae of treatment. In addition, with continued improvements in extracranial disease control and systemic therapy options, many patients are faced with the problem of recurrent intracranial disease in the setting of prior intracranial therapy.

At the present time, there are wide array of modalities employed in the treatment of oligometastatic brain metastases both in the up-front and recurrent setting, each with their own unique potential advantages and pitfalls (7). Options include surgical resection, single fraction radiosurgery, both linac-based and Gamma Knife based, WBRT, and various combinations thereof. A recent ASTRO consensus statement highlights the relative merits and adversities of these modalities, all of which are considered appropriate options in different circumstances (8). A more recent management trend with a limited but growing experience, however, is the use of hypofractionated local therapy for limited brain metastases, which has been given various designations including fractionated stereotactic radiosurgery (FSRS), hypofractionated conformal stereotactic radiation therapy (HCSRT), and hypofractionated stereotactic radiosurgery (HSRS), and has employed various modalities including linac cone, linac IMRT, linac 3D, and Cyber Knife, as well as non-invasive vs. invasive stereotactic positioning systems (9–17). Potential advantages of the hypofractionated technique, particularly for larger intracranial targets, include the patient convenience of a relatively few number of treatments, the normal tissue sparing achieved through focal irradiation, as well as the improved normal tissue tolerance of high dose radiation through fractionation (18).

In comparison to linac-based methods, the use of helical TomoTherapy (HT) is less reported for the treatment of brain metastases. HT is a unique IMRT delivery system capable of achieving highly conformal dose distributions with good coverage of intracranial targets and normal tissue sparing (19). Several reports have described the use of HT in the treatment of brain metastases both as an integrated boost within a simultaneous WBRT plan, and alone as a focal treatment (20–24). In addition, previous dosimetric investigations have indicated that HT possesses favorable dosimetric properties for the application of focal irradiation of metastatic lesions in comparison with more commonly used modalities including linac and Gamma Knife radiosurgical systems (25–27).

At our institution, patients with limited intracranial metastases or a resection cavity deemed eligible for stereotactic radiosurgery (SRS) are most commonly treated with a single fraction using Gamma Knife radiosurgery if the lesion is amenable to this type of treatment based on size and location criteria. Due to concerns of toxicity, larger target lesions (typically >3 cm diameter) may not be recommended for single fraction SRS on a provider dependent basis. In addition for recurrent lesions in the setting of prior WBRT, repeat WBRT may be avoided due to toxicity concerns by focal treatment to recurrent lesions. The use of hypofractionated radiotherapy with TomoTherapy therefore represents an option for select patients when local control of limited intracranial disease not amenable to single fraction SRS is desired while avoiding initial or repeat WBRT. The purpose of this study was to report our institutional experience with the use of HT for focal irradiation alone to intact brain metastases or the post-operative resection cavity for patients with oligometastatic disease both in the initial and recurrent setting.

## Materials and Methods

### Patient selection

Patients treated with hypofractionated TomoTherapy-based focal irradiation for brain metastases were identified for this retrospective review through a query of the departmental electronic medical record. Patients were eligible for analysis if they had a histologically documented primary malignancy of any solid tumor with confirmed brain metastases, either histologically or radiographically. All patients were treated with hypofractionated HT-based IMRT for 1–9 lesions, which include intact brain metastases or a post-operative resection cavity. Hypofractionated therapy consisted of treatments delivered to focal brain volumes only (i.e., not as a component of WBRT) with 4–10 fractions of 250–550 cGy per fraction. Two of the patients treated at 2.5 Gy per fraction had numerous target lesions (nine and four) prompting concern over the volume of brain tissue that would have been irradiated at higher dose per fraction, and one patient was intended to undergo treatment at 5 Gy per fraction but elected to proceed with treatment at 2.5 Gy per fraction. These patients were included in this analysis due to the fact that the intent of treatment was to deliver focal irradiation to specified lesions as opposed to repeat WBRT. Treatments for all patients in this study were completed between 2/2009 and 10/2013. No standard selection criteria were employed for the determination to use HT-based focal irradiation; this was practitioner dependent on a case-by-case basis. In general, however patients selected for this modality were not eligible for single fraction SRS due to size constraints of the target lesion(s). All aspects of this retrospective review were approved by the IRB.

### Radiotherapy planning and delivery

All patients were treated with HT-based IMRT on a TomoTherapy Hi-Art unit using TomoPlan treatment planning software (TomoTherapy, Madison, WI, USA). Treatment planning was based on thin slice kV CT simulation images of the brain registered to MR images acquired in the treatment position using a thermoplastic mask for immobilization. Target delineation consisted of identification of the primary lesion(s) using the MRI T1 + C sequence to identify the GTV with a 3 mm PTV expansion. The most common fractionation schemes were 25 Gy in five fractions of 500 cGy per fraction, and 27.5 Gy in five fractions of 550 cGy per fraction. All treatments were designed to deliver the prescription dose to 95% of the PTV volume. Treatments delivering more than 400 cGy per fraction were delivered on alternating days as opposed to consecutively.

### Follow up and statistical analysis

After radiotherapy completion, all patients were followed up at approximately 3-month intervals with repeat MRI imaging until death or inability to return for evaluation. During the study period, 11 patients had imaging documented in the medical record within 2 months of death or last follow up, 1 patient had an MRI scan 4.5 months prior to death, and 1 patient had an MRI scan 6 months prior to death (this patient was entered into hospice approximately 2 months after this MRI scan however lived 4 months in hospice care during which time imaging was not performed). The median time interval between the last imaging and death or last follow up was 32 days. Overall survival from the completion of radiation therapy was defined as the duration from the end of radiation to death from any cause. Intracranial progression free survival was defined as the duration from the completion of radiation to progression of disease within the brain or death. Overall survival from initial diagnosis was defined as the duration from pathological confirmation of malignancy from any site to death from any cause. Determination of progressive disease for the purposes of this study was made on the basis of formal radiologic reports of follow up MRI evaluations. If lesion progression was described as the most likely description for imaging findings this was regarded as progression even if histopathologic confirmation or additional subsequent imaging was not available. Survival analysis was performed using the Kaplan–Meier method. Statistical analysis was performed using MedCalc statistical software version 12.7 (MedCalc Software, Ostend, Belgium).

## Results

### Patient characteristics and treatment

**Figure 1:**
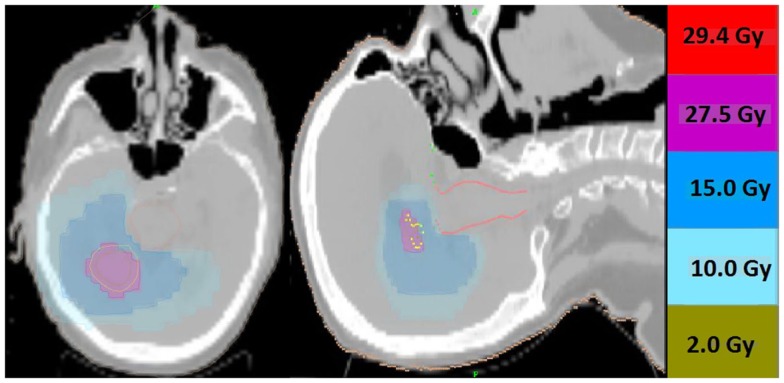
**Representative screenshots depicting isodose distributions of a plan delivered to a single intact brain metastasis with conformal avoidance of the brainstem**. The prescription does was 27.5 Gy in five fractions.

**Figure 2:**
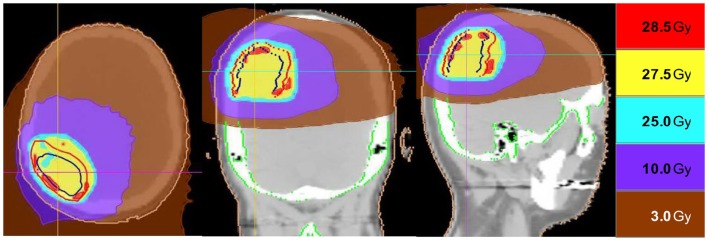
**Representative screenshots depicting isodose distributions of a plan delivered to a single post-operative resection cavity**. The prescription dose was 27.5 Gy in five fractions.

Thirteen patients were identified (7M/6F) with a median age of 62 and median KPS of 90. The majority of patients (9/13) had a single focal lesion (intact metastasis or resection cavity), and 4/13 had multiple lesions treated simultaneously. A total of 27 metastases were treated. Six patients were treated for intact lesions, six patients were treated for post-operative resection cavities, and one patient has one intact lesion and one resection cavity treated. The median and mean PTV volume was 20 and 33 cc, respectively (range 2–96 cc). The most common fractionation schemes were 25 Gy in five fractions of 500 cGy per fraction, and 27.5 Gy in five fractions of 550 cGy per fraction. One patient’s plan per patient request was modified after the first fraction from 500 cGy per fraction to 250 cGy per fraction. Histologic subtypes included colorectal (3), non-small cell lung cancer (5), renal cell carcinoma (1), breast (1), melanoma (1), uterine (1), and ovarian (1). Metastases were located in both cerebral and cerebellar locations. The majority of patients (9/13) had previously undergone intracranial radiotherapy including WBRT alone (5), WBRT + SRS (3), and SRS alone (1). Four patients had previously undergone no prior intracranial radiotherapy. Four patients were diagnosed with brain metastases concurrently with the primary tumor diagnosis, and nine patients were diagnosed with brain metastases after the initial primary tumor diagnosis. The median time from initial diagnosis to the occurrence of brain metastases was 21 months. The primary disease was stable in 7/13 patients and progressive in 6/13 patients prior to the start of XRT. The majority of patients (12/13) had at least one neurologic symptom prior to radiation including diplopia (1), headache (2), gait ataxia (2), aphasia (1), confusion and memory decline (1), weakness (3), and seizures (2). One patient was neurologically asymptomatic prior to treatment. The median follow up period after XRT was 6.2 months (range 1.26–20.13 months). Patient characteristics are summarized in Table 1. Representative plans are depicted in Figures 1 and 2.

**Table 1:** **Patient data table**.

Patient	Age	Sex	KPS	Histology	Time from Dx to BM (mo.)	Location of BM	# Lesions treated	Total dose/#Fx	Prior IC radiotherapy	Deceased
1	70	M	100	Colorectal	49	Cerebellar	1	25 Gy/5Fx	None	Yes
2	51	M	80	NSCLC	0	Multifocal	9	25 Gy/10Fx	WBRT 35 Gy	Yes
3	65	M	90	NSCLC	73	Left lateral cerebellar	1	20 Gy/5Fx	WBRT 37.5 Gy + GK SRS x2	Yes
4	60	M	100	RCC	33	Right ventricle	1	27.5 Gy/5Fx	WBRT 37.5 Gy	Yes
5	62	M	80	Melanoma	22	Multifocal	4	25 Gy/10Fx	WBRT 30 Gy	Yes
6	47	F	80	Breast	21	Right parietal	1	5 Gy × 1 + 25 Gy/10Fx	WBRT 37.5 + GK SRS x2	Unknown
7	70	F	90	NSCLC	13	Right temporal-parietal	1	27.5 Gy/5Fx	WBRT 37.5	Yes
8	83	F	90	Colorectal	61	Multifocal	3	25 Gy/5Fx	None	Yes
9	81	M	90	NSCLC	0	Right temporal-parietal	1	27.5 Gy/5Fx	GK SRS x1	Yes
10	60	M	80	NSCLC	0	Multifocal	2	25 Gy/5Fx	WBRT 30 Gy	Yes
11	55	F	100	Colon	0	Right parietal	1	27.5 Gy/5Fx	WBRT 37.5 Gy + GK SRSx4	No
12	59	F	80	Uterine adenocarcinoma	8	Right temporal	1	20 Gy/5Fx	None	Yes
13	67	F	90	Papillary serous ovarian	33	Right temporal-parietal	1	27.5 Gy/5Fx	None	No

*Dx, diagnosis; BM, brain metastases; IC, intracranial*.

### Control of intracranial disease

Intracranial in-field failure alone was exhibited in two (15%) patients, intracranial out of field failure alone was exhibited in one (8%) patient, and both in-field and out of field intracranial failure was noted in three (23%) patients. In terms of treated lesions, 8 of 27 treated lesions (29.6%) exhibited failure. Seven (54%) patients did not exhibit any intracranial failure. The median intracranial failure free survival after XRT was 6.3 months. The 6 and 12-month freedom from local failure for treated lesions was 71% and 59%.

### Overall survival

The median overall survival from initial diagnosis of malignancy was 36 months (range 8.5–55 months). The median overall survival from the completion of TomoTherapy hypofractionated XRT was 6.3 months (range 1–20 months). The 6 and 12-month OS rate was 69% and 20%. At the time of analysis, 10 patients were known to have deceased, 2 patients were known to be alive, and 1 patient was unknown status.

**Figure 3:**
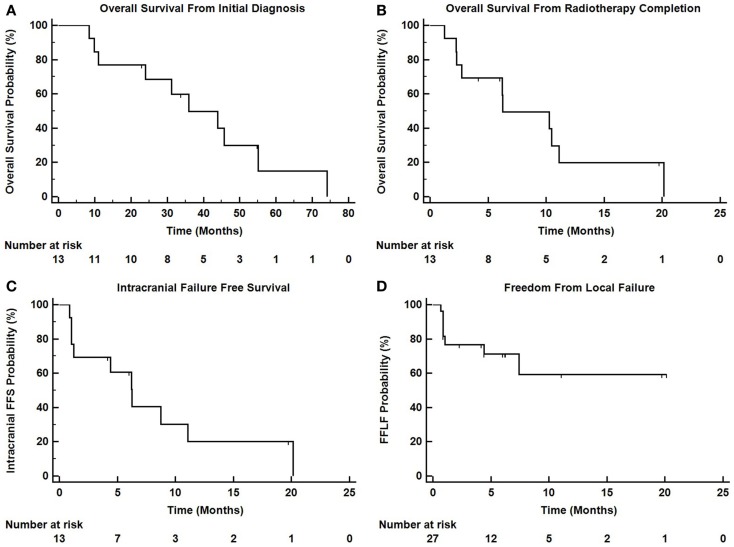
**Kaplan–Meier survival curves depicting (A) overall survival from initial diagnosis of malignancy, (B) overall survival from the completion of TomoTherapy, (C) intracranial failure free survival, and (D) freedom from local failure for treated lesions**.

Figure 3 depicts Kaplan–Meier survival curves for OS from initial diagnosis, OS from the completion of TomoTherapy, intracranial FFS, and freedom from local failure for treated lesions.

### Treatment related toxicity

Eight of 13 patients had no reported toxicities, 2 had alopecia, 1 had nausea, and 1 has nausea with headache. No >grade 3 toxicities were reported relative to the Common Terminology Criteria for Adverse Events version 4.0. One patient had hemorrhagic transformation of a treated metastasis with subsequent intracranial hemorrhage and passed away shortly thereafter.

## Discussion

Despite the increasing use of hypofractionated radiosurgical techniques in the management of brain metastases, there is a relative paucity of reports describing the use of HT for this purpose. The majority of such reports describe a treatment scheme, which incorporates a lesion specific integrated boost within a WBRT plan (20, 22, 28). Less reported however is the use of HT for hypofractionated focal irradiation alone of lesions. Sanghera et al. reported on five patients treated with HT who were unwilling or unable to undergo linac-based SRS and concluded that TomoTherapy represented a viable alternative (24). Tomita et al. reported on 16 patients with 1–4 brain metastases treated focally to 35 or 37.5 Gy in 5 fractions of whom 2 developed a local recurrence at the treated site and 3 developed new intracranial metastases (29). Nagai et al. reported on a prospective cohort of 54 patients with 128 metastases treated with a 4 fraction technique to 28 Gy using HT with an overall 1 year LC rate of 91% and a median OS of 7 months (23). In addition, dosimetric comparison studies have been performed between HT and other modalities of radiosurgical dose delivery and have established a rationale for the use of this technique. In comparison to Gamma Knife SRS, HT-based radiosurgery has been found to achieve similar target coverage and similar conformity indices, however with a smaller high dose volume but with the disadvantage of a larger amount of normal tissue irradiation (19, 25, 30). A dosimetric comparison between intensity modulated radiosurgery and HT for 3–6 brain metastases revealed equivalent conformity indices, target coverage, and sparing of the organs at risk between the modalities, however with a higher integral dose attributed to HT (26).

The present study, although not the largest experience with the use of TomoTherapy for the focal hypofractionated treatment of brain metastases, provides further support for the feasibility of this technique. The intracranial failure free survival of 54% and the median OS of 6.3 months exhibited in the present analysis are comparable to the results noted in the previous reports. Important differences however between the present study and the studies of Tomita et al. and Nagai et al. must be acknowledged. In particular, Tomita et al. included patients without any prior intracranial treatments, and Nagai et al. did not allow patients previously treated with WBRT, where as in the present study the majority of patients had received prior treatments including WBRT, SRS, or both. In addition, Nagai et al. restricted lesion size to <3 cm for eligibility whereas in the present study no such size criteria were employed. Despite these differences in patient and lesion selection criteria, the present study supports the use of HT in the treatment of lesions in the setting of prior radiotherapy or with a target size >3 cm.

Given that lesion size is frequently reported to be correlated with local control, the larger size of the target volumes treated in this series may contribute to the relatively lower local control rate (38% of patients had local failure as a component of failure, and 29.6% of the treated lesions exhibited failure) than those reported in other series. In a study of patients treated with single fraction Gamma Knife SRS, Shiau et al. reported reduced failure free survival for larger lesions, with lesions <3, 3–10, and >10 cc exhibiting 1 year local control of 87%, 63%, and 25%, respectively (31). Aoyama et al. reported that in the setting of hypofractionated SRS, local control for tumors <3 vs. >3 cc was 96% and 59%, respectively (10). Eaton et al. also reported decreased local control in the hypofractionated setting with an increase in tumor volume, and reported an overall 1 year local control of 61% and an intracranial PFS of 55% in a group of patients with a median PTV volume of 24.5 cc (12). The present series reports a similar finding of a mean PTV volume of 33 cc with 6 and 12 months freedom from local failure of 71% and 59%. A tumor with a diameter of 2 cm (radius of 1 cm) would have a volume of 4.2 cc assuming the volume is calculated according to *V* = 4/3π*r*^3^. The mean PTV volume of 33 cc in this series would correspond to a mean PTV radius of 2 cm, a diameter of 4 cm, and therefore a gross lesion diameter of >3 cm when accounting for PTV margin. Therefore, the rate of local control exhibited in this series is consistent in part with the larger size of lesion treated in comparison with other series. In addition, patient selection factors may constitute an important component of disease control outcomes, as patients treated in the recurrent setting after prior intracranial radiotherapy may represent a group with more aggressive biology at baseline. The doses per fraction used in this series are lower than those employed in some series, and may have contributed to a more modest local control. Due to toxicity concerns in a largely pre-treated cohort of patients (the majority having previously undergone WBRT), this fractionation has been the institutional preference.

Despite the possible adverse factors attributable to the patients in this series, the use of HT in the focal treatment of intracranial lesions was associated with acceptable intracranial disease control, overall survival, and toxicity when compared with other series using alternative modalities. Hypofractionated HT-based SRS should be considered a viable option in the management of brain metastases or resection cavities in the initial or recurrent setting, particularly when the size of the target is not amenable to a single fraction treatment. We propose that a fractionation schedule of 25 or 27.5 Gy in five fractions is a reasonable approach providing adequate local control and toxicity profile in the proper setting with results that are similar to those achieved by other series.

## Conflict of Interest Statement

The authors declare that they have no conflict of interest. They have no affiliations with or involvement in any organization or entity with any financial interest (such as honoraria; educational grants; participation in speakers’ bureaus; membership, employment, consultancies, stock ownership, or other equity interest; and expert testimony or patient-licensing arrangements), or non-financial interest (such as personal or professional relationships, affiliations, knowledge, or beliefs) in the subject matter or materials discussed in this manuscript.
